# On performance evaluation of NOMA-aided SIMO multi-hop schemes using energy harvesting and fountain coding based information accumulation

**DOI:** 10.1371/journal.pone.0344332

**Published:** 2026-03-27

**Authors:** Ngo Hoang An, Lam-Thanh Tu, Tran Trung Duy, Tien-Tung Nguyen

**Affiliations:** 1 Faculty of Electronics Technology, Industrial University of Ho Chi Minh, Ho Chi Minh City, Vietnam; 2 Faculty of Electrical and Electronics Technology, Ho Chi Minh City University of Industry and Trade, Ho Chi Minh City, Vietnam; 3 Advanced Intelligent Technology Research Group, Faculty of Electrical and Electronics Engineering, Ton Duc Thang University, Ho Chi Minh City, Vietnam; 4 Faculty of Telecommunications 02, Posts and Telecommunications Institute of Technology, Ho Chi Minh City, Vietnam; Guangdong University of Petrochemical Technology, CHINA

## Abstract

In this paper, we propose a new non-orthogonal multiple access (NOMA)-aided Single-Input Multiple-Output (SIMO) multi-hop relay scheme using wirelessly energy harvesting (EH) and Fountain Codes (FCs). Specifically, a single antenna source node employs NOMA to transmit two data streams to two multiple antenna destinations with the assistance of *N* intermediate multiple antenna Decode-and-Forward (DF) relays. The destinations accumulate received information until sufficient data is gathered to successfully recover the original data. This configuration ensures the SIMO structure is maintained across all hops, as the relays utilize multiple antennas for reception and a single antenna for transmission. To enhance channel capacity at the relay and destination nodes, the selection combining (SC) technique is applied. For data transmission, both the source and relay nodes harvest wireless energy from a power station deployed within the network, follows a time-switching approach. We first derive exact expressions for the end-to-end (e2e) instantaneous signal-to-interference-plus-noise ratios (SINRs) at both destinations, along with their corresponding cumulative distribution functions (CDFs) over Rayleigh fading channels. Then, we derive analytical expressions for the average channel capacity (ACC-e2e) and outage probability (OP-e2e) at each destination and validate their accuracy through Monte Carlo simulations.

## Introduction

Multi-hop relay networks [1–3] are commonly applied to self-configuring networks, such as wireless sensor networks, device-to-device communication networks, etc., without infrastructure support (or only partial support). In [4], the authors proposed and evaluated the end-to-end (e2e) performance of cluster-based multi-hop relaying networks, where each cluster selects one cluster node to receive and forward the received data from the previous cluster, using decode-and-forward (DF) technique. Similar to [4], the authors in [5] studied the e2e performance of multi-hop relay networks using node selection at each hop over Rayleigh fading channels. However, unlike [4], the study in [5] considered the impact of co-channel interference on the e2e performance. In [6], the authors evaluated performance of multi-hop relay networks, in terms of outage probability (OP) of data channels and intercept probability (IP) of eavesdropping channels. In addition, published work [6] investigated the trade-off between OP and IP, as well as the impact of hardware imperfection on the considered performance. In [7], the source and the selected relays at each cluster must adjust their transmit power to reduce the e2e IP below a desired value. Then, an optimal relay selection method at each hop is performed to improve the OP performance, in the presence of correlated data channels and multiple eavesdroppers. In [8], a multi-hop full-duplex relay scheme employing multiple-input multiple-output (MIMO) devices and short-packet communications (SPC) was proposed and analyzed. The scheme in [8] significantly improves the e2e performance in terms of throughput, energy efficiency, reliability and latency. Published work [9] evaluated the e2e block error rate and secrecy throughput of secure multi-hop Internet of Things (IoT) relay networks under the impact of imperfect channel state information (CSI). However, the multi-hop relaying schemes in [1–7] do not consider radio-frequency energy harvesting (RF-EH) as a means of powering the wireless nodes.

The RF-EH technique [10–12] can help address the limited power issue in self-configuring networks, allowing network nodes to harvest energy from wireless signals in their surrounding environment. In time-switching (TS) EH technique [13], the EH phase and the data transmission phase are performed sequentially, i.e., the transmitters first harvest RF energy and then use the harvested energy to transmit their data. In power-switching (PS) EH technique [13], receivers can simultaneously harvest energy and decode information from the received signals of transmitters. In particular, the received signals are divided into two parts: one part is used for EH, while the remaining part is used for data decoding. In [14], the authors designed a multi-hop device-to-device communication model that utilizes RF-EH and operates in an underlay cognitive environment, where the transmit power of secondary transmitters depends on the interference threshold and the energy collected from the power station. The authors of [15,16] extended the work in [14] to a cluster-based cognitive multi-hop scheme with node selection at each cluster. In addition, multiple power station and multi-antenna power station cases were considered in [15] and [16], respectively. In [17], the authors proposed a cooperative-based multi-hop transmission scheme powered by a single power station. Additionally, incremental cooperation at each hop and a partial relay selection method (see [18]) were examined in [17]. The authors of [19] studied a power station-aided multi-hop mobile-to-mobile relay scenario using cooperative communication at each hop, where all nodes were mobile and the channels were cascaded Rayleigh fading. Unlike [14–17] and [19], this paper applies non-orthogonal multiple access (NOMA) to the RF EH–aided multi-hop relaying scheme to improve system throughput, compared with the corresponding scheme without using NOMA.

The NOMA technique [20–22] enables a transmitter to send different data to multiple receivers at the same time, frequency and code. To achieve this, a power allocation method for the transmitted signals is performed at the transmitter, while a successive interference cancellation (SIC) method is performed at the receivers. Consequently, NOMA significantly enhances throughput and multiplexing gain, as compared with the conventional orthogonal multiple access (OMA) techniques. Recently, there have been few published works, in which NOMA has been applied to multi-hop relay networks to improve the e2e system throughput. In [23], the authors evaluated the OP-IP trade-off for secure multi-hop communication networks using NOMA, in the presence of an active eavesdropper. In particular, the source and relay nodes in [23] must reduce the transmit power to ensure that the instantaneous signal-to-noise ratio (SNR) received by the eavesdropper remains below a predetermined threshold. Additionally, the transmitters in [23] use NOMA at each hop to forward two data to a destination node. Published work [24] compared the e2e OP and the e2e throughput of multi-hop relay models using NOMA with those of the corresponding OMA models over Nakagami-m fading channels, considering the impact of imperfect transceiver hardware. In [25], the secrecy outage probability of NOMA-based multi-hop transmission schemes was evaluated through analysis and simulations. However, this paper considers Fountain codes (FCs) and the RF-EH technique, which are not investigated in [23–25].

Fountain codes (FCs), also known as rateless codes [26,27], have gained significant interest because of their ease of implementation and flexibility in varying channel environments. With this coding technique, the transmitter can produce and send an endless sequence of encoded packets. As long as the receiver collects a sufficient number of information (i.e., more than the amount of information originally sent), the original message can be fully reconstructed [26,27]. This characteristic eliminates the dependency on a feedback link and removes the need for receivers to possess channel state information (CSI). In the studies [28,29], several approaches integrating FCs into cooperative relay schemes have been introduced. These schemes have demonstrated improvements in both system capacity and transmission efficiency, outperforming traditional cooperative methods. Reference [30] explored the use of FCs in broadcasting over cognitive radio networks. The findings indicated that the proposed scheme in [30] notably decreased transmission delays in comparison to conventional methods that do not utilize FCs. In [31], the authors introduced a dynamic cooperative transmission algorithm that leverages mutual information accumulation, where the use of FCs allows nodes to mitigate retransmission delays under poor channel conditions. In addition, mutual information accumulation accelerates data collection at the receiver, helping to further minimize transmission delay. The authors of [32] investigated a dual-hop cooperative transmission system powered by RF-EH, where dynamic DF relaying was utilized in combination with FCs. In [33], the authors analyzed decoding failure probability in multi-hop relay networks employing SPC, considering both recoding and e2e FC techniques. Reference [34] proposed an FC-based multi-path routing algorithm designed for low-latency and highly dynamic unmanned aerial vehicle (UAV) networks. In [35], performance of cooperative full-duplex (FD) NOMA relay scheme using FCs and Intelligent Reflective Surface (IRS) was performed.

Unlike the previous works, this paper investigates the performance of single-input multiple-output (SIMO) multi-hop relay systems employing FCs, NOMA and RF-EH. Although the authors of [17] also investigated the multi-hop relaying scheme using FCs and RF-EH, their approach employed cooperative transmission with relay selection at each hop, and did not employ the NOMA technique or the SIMO model. In [23–25], NOMA-based multi-hop relay schemes were proposed. However, these works did not take into account FCs, RF-EH, and SIMO. Different from [31,32], our scheme considers the multi-hop relay scenario which integrates NOMA and RF-EH. While [33] and [34] focus on the FC-based multi-hop relaying schemes operating in SPC and UAV environments, respectively, this paper investigates such schemes in the NOMA, RF-EH, and SIMO environment. Although the published work [36] proposed a joint FC- and NOMA-based relay scheme combining with the advanced techniques such as IRS and FD, it did not consider the multi-hop relaying model or the SIMO model.

In the proposed scheme, a source node uses NOMA to simultaneously transmits two data to two intended destinations, via the help of DF relays. Each destination accumulates received information until it becomes sufficient to reconstruct the desired data. To improve the channel capacity at both the relay and destination nodes, the selection combining (SC) technique is also employed. Furthermore, the source and relay nodes harvest wireless energy from a power station deployed within the network for the data transmission, follows a time-switching approach.

Next, the main contributions of this paper can be listed as follows:

- Firstly, we propose the new NOMA-Aided SIMO Multi-hop Schemes Using RF-EH and FCs, which obtains higher throughput, energy efficiency and reliable communication.
- Secondly, we derive exact closed-form expressions for the e2e instantaneous signal-to-interference-plus-noise ratios (SINRs) at both destinations and their corresponding cumulative distribution functions (CDFs) under Rayleigh fading channels.
- Thirdly, from the derived CDFs of SINRS, we then give analytical formulas for the e2e average channel capacity (ACC-e2e) and outage probability (OP-e2e) at each destination. All the derived formulas are then validated through Monte Carlo simulations.
- Finally, simulation and theoretical results demonstrate the effectiveness of the proposed scheme and provide insights into the impact of key system parameters.

## System model

**Fig 1:**
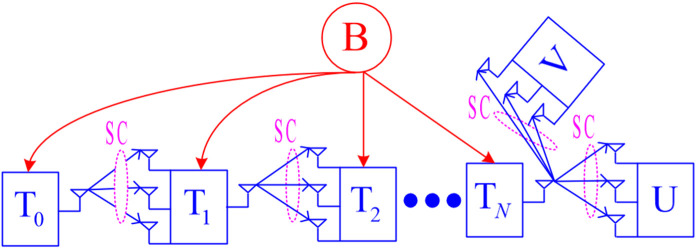
System model of the proposed NOMA-aided multi-hop relay schemes employing FCs and RF-EH.

Fig 1 illustrates the system model of NOMA-aided multi-hop relay schemes employing FCs and RF-EH, which includes one transmitter (*T*_0_), *N* relays ($T_{n},n=1,2,...,N$), and one power station (B). In this figure, the source node $\left(T_{0}\right)$ attempts to transmit the data $p_{U}$ and $p_{V}$ to its destinations U and V, respectively. Assume that data transmission is carried out with the assistance of intermediate DF relays denoted by $T_{n}$. For the transmitters including $T_{0}$ and $T_{n}$, they harvest energy from the single-antenna power station (B), and utilize the harvested energy to transmit the data. Assume that all the transmitters are equipped with one transmit antenna, while the receivers such as $T_{n}$, U and V have *M* receive antennas and use the SC technique. It is worth noting that the implementation of SC is simpler than that of maximal ratio combining (MRC), because SC only requires selecting the branch with the highest SNR, while MRC requires a combination of all the branches, leading to higher computational and hardware complexity. Furthermore, the implementation of SIMO schemes is much simpler than that of MIMO schemes. This is because MIMO schemes require perfect CSI at both the transmitter and the receiver, which incurs significant latency and computational overhead. Assume that the e2e transmission time of $p_{U}$ and $p_{V}$ is one time unit, and hence, the time allocated for each hop (each time slot) is τ=1/(N+1).

Let $\overset{\left(m\right)}{\underset{\text{AC}}{\gamma }}$ denote the channel gain between the transmitter $\text{A}\in \{T_{0},T_{n}\}$ and the *m*-th antenna of the receiver $\text{C}\in \{T_{n},\text{U},\text{V}\}$, where *m* = 1,2,...,*M*. All channels are assumed to experience quasi-static, frequency-flat Rayleigh fading. As given in [23,24], $\gamma_{AC^{\left(m\right)}}$ is an exponential random variable, and its CDF and probability density function (PDF) are given, respectively as


$$F_{\gamma_{AC^{\left(m\right)}}}\left(x\right)=1-\exp\left(-\lambda_{AC}x\right),f_{\gamma_{AC^{\left(m\right)}}}\left(x\right)=\lambda_{AC}\exp\left(-\lambda_{AC}x\right),$$ (1)

where $\lambda_{AC}={\left(d_{AC}\right)}^{\beta }$ with $d_{AC}$ being distance between A and C, β is a path-loss exponent [23,24].

Considering the nth time slot, the relay $T_{n-1}$ uses energy collected from the Power Station (B) based on the Time-Switching (TS) protocol to send the data to $T_{n}$. At first, $T_{n-1}$ harvests energy from B during the time ατ, where α(0<α<1) is a predetermined value [18,19]. Then, the energy harvested by $T_{n-1}$ can be given as (In this paper, we use the linear EH model [18,19]).


$$E_{T_{n-1}}=\eta \alpha \tau P_{B}\gamma_{BT_{n-1}},$$ (2)

where η(0<η<1) is conversion efficiency [18,19], and $P_{B}$ is transmit power of the power station (B).

The remaining time (1−α)τ is used for the data transmission between $T_{n-1}$ and $T_{n}$, and hence the transmit power of $T_{n-1}$ can be formulated as


$$P_{T_{n-1}}=\frac{E_{T_{n}}}{\left(1-\alpha \right)\tau }=\chi P_{B}\gamma_{BT_{n-1}},$$ (3)

where *χ* = *ηα* / ( 1 − *α* ) .

Using NOMA, $T_{n-1}$ combines modulated signals of the data $p_{U}$ and $p_{V}$ as in [36]: $p_{+}=\sqrt{a_{1}P_{T_{n-1}}}p_{U}+\sqrt{a_{2}P_{T_{n-1}}}p_{V}$, where $a_{1}+a_{2}=1$. Additionally, we assume that U is nearer to $T_{N}$ than V, we can then set *a*_1_ and *a*_2_ as in [36]: $a_{2}>a_{1}$. It is noted that the values of *a*_1_ and *a*_2_ are used at all the hops. We also note adaptive power allocation across hops may further improve the system performance, and extending the proposed model to incorporate such adaptive power allocation is straightforward.

Next, $T_{n-1}$ sends *p*_+_ to $T_{n}$ which decodes $p_{V}$ first, and then performs SIC to remove $p_{V}$ before decoding $p_{U}$ [36–40]. The SINRs obtained at the *m*th antenna of $T_{n}$ for decoding $p_{V}$ and $p_{U}$ can be formulated, respectively, as in [36–40]:


$$\begin{array}{cc} \psi_{T_{n-1}T_{n}^{\left(m\right)}}^{p_{V}} & =\frac{a_{2}P_{T_{n-1}}\gamma_{T_{n-1}T_{n}^{\left(m\right)}}}{a_{1}P_{T_{n-1}}\gamma_{T_{n-1}T_{n}^{\left(m\right)}}+\sigma_{0}^{2}}=\frac{a_{2}\chi \Delta_{B}\gamma_{BT_{n-1}}\gamma_{T_{n-1}T_{n}^{\left(m\right)}}}{a_{1}\chi \Delta_{B}\gamma_{BT_{n-1}}\gamma_{T_{n-1}T_{n}^{\left(m\right)}}+1},\\ \psi_{T_{n-1}T_{n}^{\left(m\right)}}^{p_{U}} & =a_{1}\chi \Delta_{B}\gamma_{BT_{n-1}}\gamma_{T_{n-1}T_{n}^{\left(m\right)}}, \end{array}$$ (4)

where $\Delta_{B}=P_{B}/\sigma_{0}^{2}$.

It is worth noting from (4) that $T_{n}$ can know the value of the channel gains $\gamma_{T_{n-1}\overset{\left(m\right)}{\underset{n}{T}}}$ through channel estimation, but cannot know the value of $\gamma_{BT_{n-1}}$. Therefore, $T_{n}$ has to select its receive antenna so that the value of $\gamma_{T_{n-1}\overset{\left(m\right)}{\underset{n}{T}}}$ is highest, i.e.,


$$b:\gamma_{T_{n-1}T_{n}^{\left(b\right)}}=\underset{m=1,2,...,M}{\max}\left(\gamma_{T_{n-1}T_{n}^{\left(m\right)}}\right),$$ (5)

where *b* denotes the selected antenna of $T_{n}$.

As a result, the SINRs obtained at $T_{n}$ for decoding $p_{V}$ and $p_{U}$ can be expressed, respectively as


$$\begin{array}{cc} \psi_{T_{n-1}T_{n}^{\left(b\right)}}^{p_{V}} & =\frac{a_{2}\chi \Delta_{B}\gamma_{BT_{n-1}}\gamma_{T_{n-1}T_{n}^{\left(b\right)}}}{a_{1}\chi \Delta_{B}\gamma_{BT_{n-1}}\gamma_{T_{n-1}T_{n}^{\left(b\right)}}+1},\\ \psi_{T_{n-1}T_{n}^{\left(b\right)}}^{p_{U}} & =a_{1}\chi \Delta_{B}\gamma_{BT_{n-1}}\gamma_{T_{n-1}T_{n}^{\left(b\right)}}. \end{array}$$ (6)

Similarly, we can formulate the SINRs obtained at the destination U for decoding $p_{V}$ and $p_{U}$, respectively as


$$\begin{array}{cc} \overset{p_{V}}{\underset{T_{N}U^{\left(b\right)}}{\psi }} & =\frac{a_{2}\chi \Delta_{B}\gamma_{BT_{N}}\gamma_{T_{N}U^{\left(b\right)}}}{a_{1}\chi \Delta_{B}\gamma_{BT_{N}}\gamma_{T_{N}U^{\left(b\right)}}+1},\\ \overset{p_{U}}{\underset{T_{N}U^{\left(b\right)}}{\psi }} & =a_{1}\chi \Delta_{B}\gamma_{BT_{N}}\gamma_{T_{N}U^{\left(b\right)}}, \end{array}$$ (7)

where $b:\gamma_{T_{N}U^{\left(b\right)}}=\underset{m=1,2,...,M}{\max}\left(\gamma_{T_{N}U^{\left(m\right)}}\right)$.

For the destination V, it directly decodes its data $p_{V}$, and the obtained SINR can be given as


$$\overset{p_{V}}{\underset{T_{N}V^{\left(b\right)}}{\psi }}=\frac{a_{2}\chi \Delta_{B}\gamma_{BT_{N}}\gamma_{T_{N}V^{\left(b\right)}}}{a_{1}\chi \Delta_{B}\gamma_{BT_{N}}\gamma_{T_{N}V^{\left(b\right)}}+1},$$ (8)

where $b:\gamma_{T_{N}V^{\left(b\right)}}=\underset{m=1,2,...,M}{\max}\left(\gamma_{T_{N}V^{\left(m\right)}}\right)$.

Since the channel gains $\gamma_{XY^{\left(m\right)}}$ are assumed to be independent and identically distributed random variables, CDFs and PDFs of $\gamma_{XY^{\left(b\right)}}$ can be expressed under the following forms (see [15]):


$$\begin{array}{cc} F_{\gamma_{XY^{\left(b\right)}}}\left(x\right) & =\mathrm{Pr}\left(\underset{m=1,2,...,M}{\max}\left(\gamma_{XY^{\left(m\right)}}\right)<x\right)={\left[F_{\gamma_{XY^{\left(m\right)}}}\left(x\right)\right]}^{M}\\ & ={\left(1-\exp\left(-\lambda_{XY}x\right)\right)}^{M},\\ f_{\gamma_{XY^{\left(b\right)}}}\left(x\right) & =M\lambda_{XY}\exp\left(-\lambda_{XY}x\right){\left(1-\exp\left(-\lambda_{XY}x\right)\right)}^{M-1}\\ & =\sum_{w=0}^{M-1}{\left(-1\right)}^{w}\overset{w}{\underset{M-1}{C}}M\lambda_{XY}\exp\left(-\left(w+1\right)\lambda_{XY}x\right), \end{array}$$ (9)

where $\gamma_{XY^{\left(b\right)}}\in \left\{\gamma_{T_{n-1}\overset{\left(b\right)}{\underset{n}{T}}},\gamma_{T_{N}V^{\left(b\right)}},\gamma_{T_{N}U^{\left(b\right)}}\right\}$, $\lambda_{XY}\in \left\{\lambda_{T_{n-1}T_{n}},\lambda_{T_{N}V},\lambda_{T_{N}U}\right\}$ and $\overset{r}{\underset{M}{C}}$ denotes a binomial coefficient, i.e.,


$$\overset{r}{\underset{M}{C}}=\frac{M!}{r!\left(M-r\right)!}.$$ (10)

Due to the DF relaying technique [23,24], we can formulate the SINRs obtained at V and U, respectively as


$$\psi_{V}^{e2e}=\min\left(\underset{n=1,2,...,N}{\min}\left(\psi_{T_{n-1}T_{n}^{\left(b\right)}}^{p_{V}}\right),\psi_{T_{N}V^{\left(b\right)}}^{p_{V}}\right),$$ (11)

$$\begin{array}{cc} \psi_{U}^{e2e} & =\min\left(\underset{n=1,2,...,N}{\min}\left(\psi_{T_{n-1}T_{n}^{\left(b\right)}}^{p_{V}},\psi_{T_{n-1}T_{n}^{\left(b\right)}}^{p_{U}}\right),\min\left(\psi_{T_{N}U^{\left(b\right)}}^{p_{V}},\psi_{T_{N}U^{\left(b\right)}}^{p_{U}}\right)\right)\\ & =\min\left(\underset{n=1,2,...,N}{\min}\left(\psi_{T_{n-1}T_{n}}^{\min}\right),\psi_{T_{N}U}^{\min}\right), \end{array}$$ (12)

where


$$\begin{array}{cc} \psi_{T_{n-1}T_{n}}^{\min} & =\min\left(\psi_{T_{n-1}T_{n}^{\left(b\right)}}^{p_{V}},\psi_{T_{n-1}T_{n}^{\left(b\right)}}^{p_{U}}\right),\\ \psi_{T_{N}U}^{\min} & =\min\left(\psi_{T_{N}U^{\left(b\right)}}^{p_{V}},\psi_{T_{N}U^{\left(b\right)}}^{p_{U}}\right). \end{array}$$ (13)

Finally, we can formulate the e2e instantaneous channel capacity obtained at V and U, respectively as in [14,15]:


$$\begin{array}{cc} \overset{V}{\underset{e2e}{C}} & =\left(1-\alpha \right)\tau \log_{2}\left(1+\overset{e2e}{\underset{V}{\psi }}\right),\\ \overset{U}{\underset{e2e}{C}} & =\left(1-\alpha \right)\tau \log_{2}\left(1+\overset{e2e}{\underset{U}{\psi }}\right). \end{array}$$ (14)

Let us denote ${ℐ}_{req}$ as the required information that U and V need to accumulate in order to recover their desired data. Similar to [28], the required time for U and V to sufficiently receive amount of information ${ℐ}_{req}$ can be calculated, respectively as


$$\begin{array}{cc} \overset{U}{\underset{e2e}{𝒯}} & =\frac{{ℐ}_{req}}{\overset{U}{\underset{e2e}{C}}}=\frac{{ℐ}_{req}}{\left(1-\alpha \right)\tau \log_{2}\left(1+\overset{e2e}{\underset{U}{\psi }}\right)},\\ \overset{V}{\underset{e2e}{𝒯}} & =\frac{{ℐ}_{req}}{\overset{V}{\underset{e2e}{C}}}=\frac{{ℐ}_{req}}{\left(1-\alpha \right)\tau \log_{2}\left(1+\overset{e2e}{\underset{V}{\psi }}\right)}. \end{array}$$ (15)

## Performance analysis

This section analyzes the OP-e2e and ACC-e2e at each destination. The OP-e2e at U(V) represents the probability that the information accumulation time ${𝒯}_{U}\left({𝒯}_{V}\right)$ exceeds a maximum delay threshold ${𝒯}_{\max}$, i.e.,


$$\overset{X}{\underset{e2e}{OP}}=\mathrm{Pr}\left(\overset{X}{\underset{e2e}{𝒯}}>{𝒯}_{\max}\right),$$ (16)

where X∈{U,V}.

Then, the ACC-e2e obtained at the destination X can be expressed as


$$\overset{X}{\underset{e2e}{ACC}}=ℰ\left\{\overset{X}{\underset{e2e}{C}}\right\},$$ (17)

where ℰ{.} denotes an expected operator.

We now attempt to derive expressions of CDFs of $\overset{e2e}{\underset{V}{\psi }}$ and $\overset{e2e}{\underset{U}{\psi }}$.

CDF of $\overset{e2e}{\underset{V}{\psi }}$ and $\overset{e2e}{\underset{U}{\psi }}$

From (11), we can formulate CDF of $\overset{e2e}{\underset{V}{\psi }}$ as


$$\begin{array}{cc} F_{\overset{e2e}{\underset{V}{\psi }}}\left(x\right) & =\mathrm{Pr}\left(\min\left(\underset{n=1,2,...,N}{\min}\left(\overset{p_{V}}{\underset{T_{n-1}\overset{\left(b\right)}{\underset{n}{T}}}{\psi }}\right),\overset{p_{V}}{\underset{T_{N}V^{\left(b\right)}}{\psi }}\right)<x\right)\\ & =1-\left(1-F_{\overset{p_{V}}{\underset{T_{N}V^{\left(b\right)}}{\psi }}}\left(x\right)\right)\prod_{n=1}^{N}\left(1-F_{\overset{p_{V}}{\underset{T_{n-1}\overset{\left(b\right)}{\underset{n}{T}}}{\psi }}}\left(x\right)\right). \end{array}$$ (18)

In (18), $F_{\overset{p_{V}}{\underset{T_{n-1}\overset{\left(b\right)}{\underset{n}{T}}}{\psi }}}\left(x\right)$ can be rewritten from (6) as


$$\begin{array}{cc} F_{\overset{p_{V}}{\underset{T_{n-1}\overset{\left(b\right)}{\underset{n}{T}}}{\psi }}}\left(x\right) & =\mathrm{Pr}\left(\frac{a_{2}\chi \Delta_{B}\gamma_{BT_{n-1}}\gamma_{T_{n-1}\overset{\left(b\right)}{\underset{n}{T}}}}{a_{1}\chi \Delta_{B}\gamma_{BT_{n-1}}\gamma_{T_{n-1}\overset{\left(m\right)}{\underset{n}{T}}}+1}<x\right)\\ & =\mathrm{Pr}\left(\left(a_{2}-a_{1}x\right)\chi \Delta_{B}\gamma_{BT_{n-1}}\gamma_{T_{n-1}\overset{\left(b\right)}{\underset{n}{T}}}<x\right). \end{array}$$ (19)

It is straightforward from (19) that if *x* ≥ *a*_2_ / *a*_1_, then $F_{\overset{p_{V}}{\underset{T_{n-1}\overset{\left(b\right)}{\underset{n}{T}}}{\psi }}}\left(x\right)=1$.

We now consider the case where 0 < *x* < *a*_2_ / *a*_1_, (19) can be expressed as


$$\begin{array}{cc} F_{\overset{p_{V}}{\underset{T_{n-1}\overset{\left(b\right)}{\underset{n}{T}}}{\psi }}}\left(x\right) & =\mathrm{Pr}\left(\gamma_{BT_{n-1}}\gamma_{T_{n-1}\overset{\left(b\right)}{\underset{n}{T}}}<\theta \left(x\right)\right)\\ & =\int_{0}^{+\infty }F_{\gamma_{BT_{n-1}}}\left(\frac{\theta \left(x\right)}{y}\right)f_{\gamma_{T_{n-1}\overset{\left(b\right)}{\underset{n}{T}}}}\left(y\right)dy, \end{array}$$ (20)

where $\theta \left(x\right)=\frac{x}{\left(a_{2}-a_{1}x\right)\chi \Delta_{B}}$.

Substituting $F_{\gamma_{BT_{n-1}}}\left(.\right)$ in (1) and $f_{\gamma_{T_{n-1}\overset{\left(b\right)}{\underset{n}{T}}}}\left(.\right)$ in (9) into (20), we obtain


$$\begin{array}{cc} F_{\overset{p_{V}}{\underset{T_{n-1}\overset{\left(b\right)}{\underset{n}{T}}}{\psi }}}\left(x\right)=1- & \sum_{w=0}^{M-1}{\left(-1\right)}^{w}\overset{w}{\underset{M-1}{C}}M\lambda_{T_{n-1}T_{n}}\\ & \times \int_{0}^{+\infty }\exp\left(-\lambda_{BT_{n-1}}\frac{\theta \left(x\right)}{y}\right)\exp\left(-\left(w+1\right)\lambda_{T_{n-1}T_{n}}y\right)dy. \end{array}$$ (21)

Utilizing equation (3.324.1) in [41] to solve the integral in (21), we finally obtain


$$\begin{array}{cc} F_{\overset{p_{V}}{\underset{T_{n-1}\overset{\left(b\right)}{\underset{n}{T}}}{\psi }}}\left(x\right)=1- & \sum_{w=0}^{M-1}{\left(-1\right)}^{w}\overset{w}{\underset{M-1}{C}}2M\sqrt{\frac{\lambda_{T_{n-1}T_{n}}\lambda_{BT_{n-1}}\theta \left(x\right)}{w+1}}\\ & \times {𝒦}_{1}\left(2\sqrt{\left(w+1\right)\lambda_{T_{n-1}T_{n}}\lambda_{BT_{n-1}}\theta \left(x\right)}\right), \end{array}$$ (22)

where ${𝒦}_{1}\left(.\right)$ is the first-order modified Bessel function of the second kind [41].

With the same derivation method, we can obtain CDF $F_{\overset{p_{V}}{\underset{T_{N}V^{\left(b\right)}}{\psi }}}\left(x\right)$ as


$$\begin{array}{cc} F_{\overset{p_{V}}{\underset{T_{N}V^{\left(b\right)}}{\psi }}}\left(x\right)=1- & \sum_{w=0}^{M-1}{\left(-1\right)}^{w}\overset{w}{\underset{M-1}{C}}2M\sqrt{\frac{\lambda_{BT_{N}}\lambda_{T_{N}V}\theta \left(x\right)}{w+1}}\\ & \times {𝒦}_{1}\left(2\sqrt{\left(w+1\right)\lambda_{BT_{N}}\lambda_{T_{N}V}\theta \left(x\right)}\right). \end{array}$$ (23)

By substituting (22) and (23) into (18), we obtain an exact closed-form expression for $F_{\overset{e2e}{\underset{V}{\psi }}}\left(x\right)$ under the condition 0 < *x* < *a*_2_ / *a*_1_, as follows:


$$F_{\overset{e2e}{\underset{V}{\psi }}}\left(x\right)=1-\sum_{w=0}^{M-1}\overset{V}{\underset{N}{\chi }}\left(x\right){𝒦}_{1}\left(\overset{V}{\underset{N}{\phi }}\left(x\right)\right)\prod_{n=1}^{N}\left[\sum_{w=0}^{M-1}\overset{\left(1\right)}{\underset{n}{\chi }}\left(x\right){𝒦}_{1}\left(\overset{\left(1\right)}{\underset{n}{\phi }}\left(x\right)\right)\right],$$ (24)

where


$$\begin{array}{cc} \overset{\left(1\right)}{\underset{n}{\phi }}\left(x\right) & =2\sqrt{\left(w+1\right)\lambda_{T_{n-1}T_{n}}\lambda_{BT_{n-1}}\theta \left(x\right)},\overset{V}{\underset{N}{\phi }}\left(x\right)=2\sqrt{\left(w+1\right)\lambda_{BT_{N}}\lambda_{T_{N}V}\theta \left(x\right)},\\ \overset{\left(1\right)}{\underset{n}{\chi }}\left(x\right) & ={\left(-1\right)}^{w}\overset{w}{\underset{M-1}{C}}2M\sqrt{\frac{\lambda_{T_{n-1}T_{n}}\lambda_{BT_{n-1}}\theta \left(x\right)}{w+1}},\\ \overset{V}{\underset{N}{\chi }}\left(x\right) & ={\left(-1\right)}^{w}\overset{w}{\underset{M-1}{C}}2M\sqrt{\frac{\lambda_{BT_{N}}\lambda_{T_{N}V}\theta \left(x\right)}{w+1}}. \end{array}$$ (25)

Next, we consider CDF of $\overset{e2e}{\underset{U}{\psi }}$; from (12), we have


$$F_{\overset{e2e}{\underset{U}{\psi }}}\left(x\right)=1-\left(1-F_{\overset{\min}{\underset{T_{N}U}{\psi }}}\left(x\right)\right)\prod_{n=1}^{N}\left(1-F_{\overset{\min}{\underset{T_{n-1}T_{n}}{\psi }}}\left(x\right)\right).$$ (26)

From (6) and (13), we can formulate $F_{\overset{\min}{\underset{T_{n-1}T_{n}}{\psi }}}\left(x\right)$ in (26) as


$$\begin{array}{cc} & F_{\overset{\min}{\underset{T_{n-1}T_{n}}{\psi }}}\left(x\right)=1-\mathrm{Pr}\left(\overset{p_{V}}{\underset{T_{n-1}\overset{\left(b\right)}{\underset{n}{T}}}{\psi }}\geq x,\overset{p_{U}}{\underset{T_{n-1}\overset{\left(b\right)}{\underset{n}{T}}}{\psi }}\geq x\right)\\ & =1-\mathrm{Pr}\left(\left(a_{2}-a_{1}x\right)\chi \Delta_{B}\gamma_{BT_{n-1}}\gamma_{T_{n-1}\overset{\left(b\right)}{\underset{n}{T}}}\geq x,a_{2}\chi \Delta_{B}\gamma_{BT_{n-1}}\gamma_{T_{n-1}\overset{\left(b\right)}{\underset{n}{T}}}\geq x\right). \end{array}$$ (27)

We can observe from (27) that if $x\geq a_{2}/a_{1}$, then $F_{\overset{\min}{\underset{T_{n-1}T_{n}}{\psi }}}\left(x\right)=1$.

In the case where $x<a_{2}/a_{1}$, we can rewrite (27) as


$$\begin{array}{cc} F_{\overset{\min}{\underset{T_{n-1}T_{n}}{\psi }}}\left(x\right) & =1-\mathrm{Pr}\left(\gamma_{BT_{n-1}}\gamma_{T_{n-1}\overset{\left(b\right)}{\underset{n}{T}}}\geq \theta \left(x\right),\gamma_{BT_{n-1}}\gamma_{T_{n-1}\overset{\left(b\right)}{\underset{n}{T}}}\geq \vartheta \left(x\right)\right)\\ & =1-\mathrm{Pr}\left(\gamma_{BT_{n-1}}\gamma_{T_{n-1}\overset{\left(b\right)}{\underset{n}{T}}}\geq \max\left(\theta \left(x\right),\vartheta \left(x\right)\right)\right)\\ & =\mathrm{Pr}\left(\gamma_{BT_{n-1}}\gamma_{T_{n-1}\overset{\left(b\right)}{\underset{n}{T}}}<\max\left(\theta \left(x\right),\vartheta \left(x\right)\right)\right), \end{array}$$ (28)

where $\vartheta \left(x\right)=\frac{x}{a_{1}\chi \Delta_{B}}$.

From the result obtained in (22), we can obtain $F_{\overset{\min}{\underset{T_{n-1}T_{n}}{\psi }}}\left(x\right)$ as


$$F_{\overset{\min}{\underset{T_{n-1}T_{n}}{\psi }}}\left(x\right)=\left\{ \begin{array}{c} \overset{\left(1\right)}{\underset{\overset{\min}{\underset{T_{n-1}T_{n}}{\psi }}}{F}}\left(x\right),\;if\;\frac{a_{2}}{a_{1}}-1\leq x<\frac{a_{2}}{a_{1}}\\ \overset{\left(2\right)}{\underset{\overset{\min}{\underset{T_{n-1}T_{n}}{\psi }}}{F}}\left(x\right),\;if\;0<x<\frac{a_{2}}{a_{1}}-1 \end{array}\right.$$ (29)

where


$$\begin{array}{cc} \overset{\left(1\right)}{\underset{\overset{\min}{\underset{T_{n-1}T_{n}}{\psi }}}{F}}\left(x\right) & =\sum_{w=0}^{M-1}\overset{\left(1\right)}{\underset{n}{\chi }}\left(x\right){𝒦}_{1}\left(\overset{\left(1\right)}{\underset{n}{\phi }}\left(x\right)\right),\\ \overset{\left(2\right)}{\underset{\overset{\min}{\underset{T_{n-1}T_{n}}{\psi }}}{F}}\left(x\right) & =\sum_{w=0}^{M-1}\overset{\left(2\right)}{\underset{n}{\chi }}\left(x\right){𝒦}_{1}\left(\overset{\left(2\right)}{\underset{n}{\phi }}\left(x\right)\right), \end{array}$$ (30)

with


$$\begin{array}{cc} \overset{\left(2\right)}{\underset{n}{\phi }}\left(x\right) & =2\sqrt{\left(w+1\right)\lambda_{T_{n-1}T_{n}}\lambda_{BT_{n-1}}\vartheta \left(x\right)},\\ \overset{\left(2\right)}{\underset{n}{\chi }}\left(x\right) & ={\left(-1\right)}^{w}\overset{w}{\underset{M-1}{C}}2M\sqrt{\frac{\lambda_{T_{n-1}T_{n}}\lambda_{BT_{n-1}}\vartheta \left(x\right)}{w+1}}. \end{array}$$ (31)

Similarly, we can obtain $F_{\overset{\min}{\underset{T_{N}U}{\psi }}}\left(x\right)$ in (26) as


$$F_{\overset{\min}{\underset{T_{N}U}{\psi }}}\left(x\right)=\left\{ \begin{array}{c} \overset{\left(1\right)}{\underset{\overset{\min}{\underset{T_{N}U}{\psi }}}{F}}\left(x\right),\;if\;\frac{a_{2}}{a_{1}}-1\leq x<\frac{a_{2}}{a_{1}}\\ \overset{\left(2\right)}{\underset{\overset{\min}{\underset{T_{N}U}{\psi }}}{F}}\left(x\right),\;if\;0<x<\frac{a_{2}}{a_{1}}-1 \end{array}\right.$$ (32)

where


$$\overset{\left(1\right)}{\underset{\overset{\min}{\underset{T_{N}U}{\psi }}}{F}}\left(x\right)=1-\sum_{w=0}^{M-1}\overset{U,1}{\underset{N}{\chi }}\left(x\right){𝒦}_{1}\left(\overset{U,1}{\underset{N}{\phi }}\left(x\right)\right),\;\overset{\left(2\right)}{\underset{\overset{\min}{\underset{T_{N}U}{\psi }}}{F}}\left(x\right)=1-\sum_{w=0}^{M-1}\overset{U,2}{\underset{N}{\chi }}\left(x\right){𝒦}_{1}\left(\overset{U,2}{\underset{N}{\phi }}\left(x\right)\right),$$ (33)

with


$$\begin{array}{cc} \overset{U,1}{\underset{N}{\phi }}\left(x\right) & =2\sqrt{\left(w+1\right)\lambda_{BT_{N}}\lambda_{T_{N}U}\theta \left(x\right)},\\ \overset{U,1}{\underset{N}{\chi }}\left(x\right) & ={\left(-1\right)}^{w}\overset{w}{\underset{M-1}{C}}2M\sqrt{\frac{\lambda_{BT_{N}}\lambda_{T_{N}U}\theta \left(x\right)}{w+1}},\\ \overset{U,2}{\underset{N}{\phi }}\left(x\right) & =2\sqrt{\left(w+1\right)\lambda_{BT_{N}}\lambda_{T_{N}U}\vartheta \left(x\right)},\\ \overset{U,2}{\underset{N}{\chi }}\left(x\right) & ={\left(-1\right)}^{w}\overset{w}{\underset{M-1}{C}}2M\sqrt{\frac{\lambda_{BT_{N}}\lambda_{T_{N}U}\vartheta \left(x\right)}{w+1}}. \end{array}$$ (34)

From (26)–(34), we can express $F_{\overset{e2e}{\underset{U}{\psi }}}\left(x\right)$ under the following form:


$$F_{\overset{e2e}{\underset{U}{\psi }}}\left(x\right)=\left\{ \begin{array}{c} \overset{\left(1\right)}{\underset{\overset{e2e}{\underset{U}{\psi }}}{F}}\left(x\right),\;if\;\frac{a_{2}}{a_{1}}-1\leq x<\frac{a_{2}}{a_{1}}\\ \overset{\left(2\right)}{\underset{\overset{e2e}{\underset{U}{\psi }}}{F}}\left(x\right),\;if\;0<x<\frac{a_{2}}{a_{1}}-1 \end{array}\right.$$ (35)

where


$$\begin{array}{cc} \overset{\left(1\right)}{\underset{\overset{e2e}{\underset{U}{\psi }}}{F}}\left(x\right) & =1-\left[\sum_{w=0}^{M-1}\overset{U,1}{\underset{N}{\chi }}\left(x\right){𝒦}_{1}\left(\overset{U,1}{\underset{N}{\phi }}\left(x\right)\right)\right]\prod_{n=1}^{N}\left[\sum_{w=0}^{M-1}\overset{\left(1\right)}{\underset{n}{\chi }}\left(x\right){𝒦}_{1}\left(\overset{\left(1\right)}{\underset{n}{\phi }}\left(x\right)\right)\right],\\ \overset{\left(2\right)}{\underset{\overset{e2e}{\underset{U}{\psi }}}{F}}\left(x\right) & =1-\left[\sum_{w=0}^{M-1}\overset{U,2}{\underset{N}{\chi }}\left(x\right){𝒦}_{1}\left(\overset{U,2}{\underset{N}{\phi }}\left(x\right)\right)\right]\prod_{n=1}^{N}\left[\sum_{w=0}^{M-1}\overset{\left(2\right)}{\underset{n}{\chi }}\left(x\right){𝒦}_{1}\left(\overset{\left(2\right)}{\underset{n}{\phi }}\left(x\right)\right)\right]. \end{array}$$ (36)

### OP-e2e and ACC-e2e

From (15) and (16), the OP-e2e at the destination X (X∈{U,V}) can be formulated as


$$\overset{X}{\underset{e2e}{OP}}=\mathrm{Pr}\left(\overset{e2e}{\underset{X}{\psi }}<\rho_{th}\right)=F_{\overset{e2e}{\underset{X}{\psi }}}\left(\rho_{th}\right),$$ (37)

where


$$\rho_{th}=2^{\frac{{ℐ}_{req}}{\left(1-\alpha \right)\tau {𝒯}_{\max}}}-1.$$ (38)

Substituting (24) and (35)-(36) into (37), we obtain exact closed-form expressions of the OP-e2e at the destinations V and U, respectively.

From (17), the ACC-e2e obtained at the destination X can be expressed as


$$\overset{X}{\underset{e2e}{ACC}}=\int_{0}^{+\infty }\left(1-\alpha \right)\tau \log_{2}\left(1+x\right)f_{\overset{e2e}{\underset{X}{\psi }}}\left(x\right)dx,$$ (39)

Furthermore, using integration by parts, the ACC-e2e in (39) can be computed by using $F_{\overset{e2e}{\underset{X}{\psi }}}\left(x\right)$ as follows:


$$\begin{array}{cc} {ACC}_{e2e}^{V} & =\frac{\left(1-\alpha \right)\tau }{\ln\left(2\right)}\int_{0}^{\frac{a_{2}}{a_{1}}}\frac{1-F_{\psi_{V}^{e2e}}\left(x\right)}{1+x}dx,\\ {ACC}_{e2e}^{U} & =\frac{\left(1-\alpha \right)\tau }{\ln\left(2\right)}\left[\int_{0}^{\frac{a_{2}}{a_{1}}-1}\frac{1-F_{\psi_{U}^{e2e}}^{\left(2\right)}\left(x\right)}{1+x}dx+\int_{\frac{a_{2}}{a_{1}}-1}^{\frac{a_{2}}{a_{1}}}\frac{1-F_{\psi_{U}^{e2e}}^{\left(1\right)}\left(x\right)}{1+x}dx\right]. \end{array}$$ (40)

It is worth noting from (40) that obtaining a closed-form expression for $\overset{X}{\underset{e2e}{ACC}}$ is impossible. Therefore, we apply the Chebyshev-Gauss quadrature method to approximate it. Indeed, the Chebyshev-Gauss approximation can be stated as in [42]:


$$\int_{a}^{b}g\left(x\right)dx\approx \frac{\left(b-a\right)\pi }{2𝒩}\sum_{n=1}^{𝒩}\sqrt{1-\overset{2}{\underset{n}{y}}}g\left(x_{n}\right),$$ (41)

where 𝒩 is a designed value [42], and


$$y_{n}=\cos\left(\frac{\left(2n-1\right)\pi }{2𝒩}\right),x_{n}=\frac{b-a}{2}y_{n}+\frac{b+a}{2}.$$ (42)

Applying (41) for (40), we can approximate $\overset{X}{\underset{e2e}{ACC}}$ as follows:


$$\begin{array}{cc} \overset{V}{\underset{e2e}{ACC}} & \approx \frac{\left(1-\alpha \right)\tau \pi }{\ln\left(2\right)}\sum_{n=1}^{𝒩}\frac{a_{2}\sqrt{1-\overset{2}{\underset{n}{y}}}}{2a_{1}𝒩}G_{1}\left(x_{1,n}\right),\\ \overset{U}{\underset{e2e}{ACC}} & \approx \frac{\left(1-\alpha \right)\tau \pi }{\ln\left(2\right)}\left\{\sum_{n=1}^{𝒩}\frac{\sqrt{1-\overset{2}{\underset{n}{y}}}}{2𝒩}\left[\frac{a_{2}-a_{1}}{a_{1}}G_{2}\left(x_{2,n}\right)+G_{3}\left(x_{3,n}\right)\right]\right\}, \end{array}$$ (43)

where


$$\begin{array}{cc} G_{1}\left(x\right) & =\frac{1-F_{\overset{e2e}{\underset{V}{\psi }}}\left(x\right)}{1+x},G_{2}\left(x\right)=\frac{1-\overset{\left(2\right)}{\underset{\overset{e2e}{\underset{U}{\psi }}}{F}}\left(x\right)}{1+x},G_{3}\left(x\right)=\frac{1-\overset{\left(1\right)}{\underset{\overset{e2e}{\underset{U}{\psi }}}{F}}\left(x\right)}{1+x},\\ y_{n} & =\cos\left(\frac{(2n-1)\pi }{2𝒩}\right),x_{1,n}=\frac{a_{2}}{2a_{1}}y_{n}+\frac{a_{2}}{2a_{1}},\\ x_{2,n} & =\frac{a_{2}-a_{1}}{2a_{1}}y_{n}+\frac{a_{2}-a_{1}}{2a_{1}},x_{3,n}=\frac{1}{2}y_{n}+\frac{2a_{2}-a_{1}}{2a_{1}}. \end{array}$$ (44)

The computational cost for evaluating the analytical expressions for $\overset{X}{\underset{e2e}{ACC}}$ relies on the efficient Chebyshev-Gauss Quadrature method, guaranteeing accurate numerical results with a deterministic complexity significantly lower than Monte Carlo simulation.

## Numerical results

This section presents simulation results (Sim) to validate the theoretical analysis (Theory). In the simulation setup, the $T_{n}$ nodes are placed on a straight line as in [6,7,15–17], i.e., $T_{n}\left(n/\left(N+1\right),0\right)$, while the B, U and V nodes are positioned as B(0.5,0.5), U(1,0) and V(1,0.25), where *n* = 0,1,...,*N*.

To analyze the impact of the important parameters such as the transmit power of the power station (or the transmit SNR $\Delta_{B}$), the time allocation factor (α), the power allocation coefficient (*a*_1_ or *a*_2_), and the number of hops (*N*), values of the remaining parameters are fixed as in Table 1. It is worth noting that the derived formulas in this paper can be applied to any practical values of the parameters.

**Table 1:** System parameters and their values.

Notation	Meaning	Value
β	Path-loss exponential	3
$\overset{2}{\underset{0}{\sigma }}$	Variance of Gaussian noise	1
η	Conversion efficiency	0.25
${ℐ}_{req}$	Required amount of information	0.1
${𝒯}_{max}$	Maximum delay threshold	1.5
𝒩=500	Number of terms in Chebyshev-Gauss approximation	500

**Fig 2:**
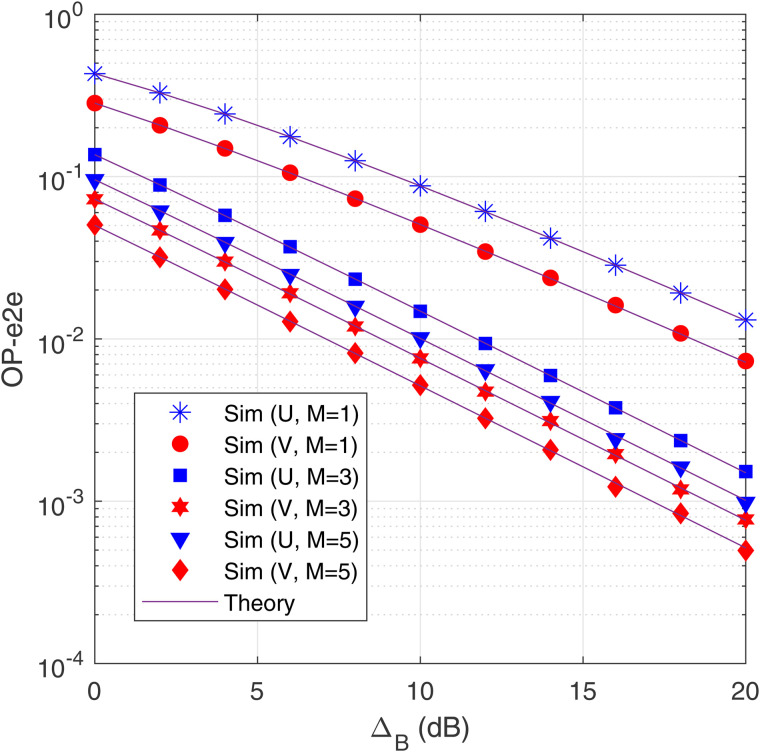
OP-e2e as a function of Δ_B_ (dB) with *N* = 3, *α* = 0.25 and *α*_1_ = 0.25.

Fig 2 illustrates the OP-e2e at the destinations U and V as a function of the transmit SNR ($\Delta_{B}$ in dB) with different values of *M*, and with *N* = 3, α=0.25 and *a*_1_ = 0.25. As observed, the OP-e2e at U and V decreases as $\Delta_{B}$ increases due to the increase in transmit power at all the transmitters. It is also seen that the OP performance of V is better than that of U. It is due to the fact that the data $p_{V}$ is allocated more transmit power than the data $p_{U}$ (i.e., $a_{2}>a_{1}$). Next, Fig 2 shows that the OP-e2e at both U and V significantly decreases with an increasing number of receiving antennas (*M*). It is because increasing *M* also improves the quality of the data channel at each hop. Because the scheme with *M* = 1 is a SISO (Single Input Single Output) configuration, Fig 2 presents that the proposed SIMO model obtains much better OP performance, as compared with the corresponding SISO one. Finally, we can see the ‘Sim’ and ‘Theory’ results match very well, which validates the correction of the derived expressions of OP-e2e.

**Fig 3:**
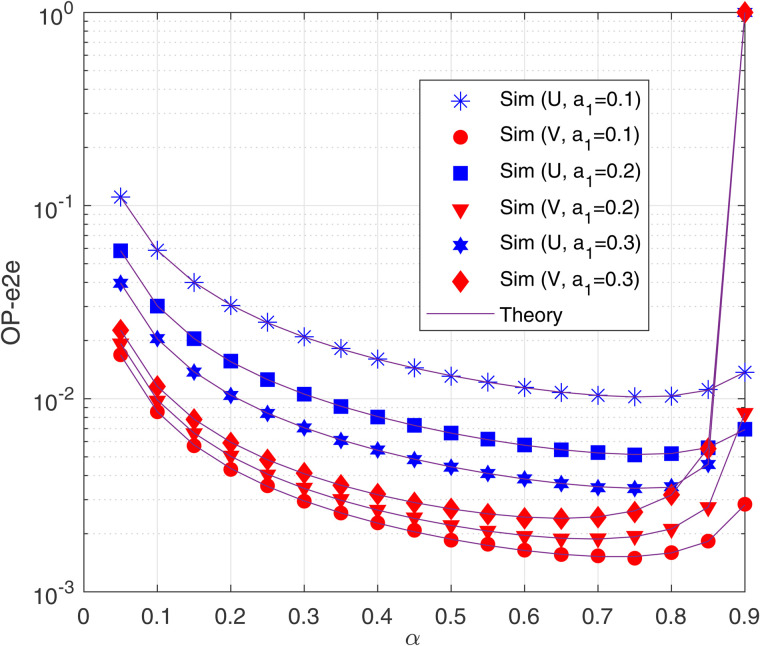
OP-e2e as a function of *α* with Δ_B_ = 15 (dB), *M* = 2 and *N* = 2.

Fig 3 investigates the impact of the power allocation factor α on the OP performance at the two destinations for various values of *a*_1_ with $\Delta_{B}$ = 15 (dB), *M* = 2 and *N* = 2. Similar to Figs 2, 3 shows that OP-e2e at V is lower than that at U. As shown in Fig 3, the OP-e2e at V (U) increases (decreases) as *a*_1_ increases from 0.1 to 0.3. This occurs because increasing *a*_1_ corresponds to allocating more (less) transmit power to the data $p_{U}$ ($p_{V}$). As a result, the smaller the value of *a*_1_, the larger the performance gap between U and V, and vice verse. Fig 3 also illustrates that α effects on the OP-e2e at U and V. Interestingly, there exist optimal values of α at which the OP-e2e at U and V is lowest. For example, with *a*_1_ = 0.2, the OP-e2e at U and V is minimized at α=0.75 and α=0.65, respectively. Again, it can be observed from Fig 3 that the ‘Sim’ and ‘Theory’ results are in a good agreement.

Here, this raises the question of what the optimal value of α is. Indeed, to determine the optimal value of α and other system parameters, we should aim to minimize the overall OP-e2e of the proposed scheme. One possible approach is to consider the average OP-e2e between nodes U and V, i.e., ${OP}_{e2e}^{avg}=\left({OP}_{e2e}^{U}+{OP}_{e2e}^{V}\right)/2$.

**Fig 4:**
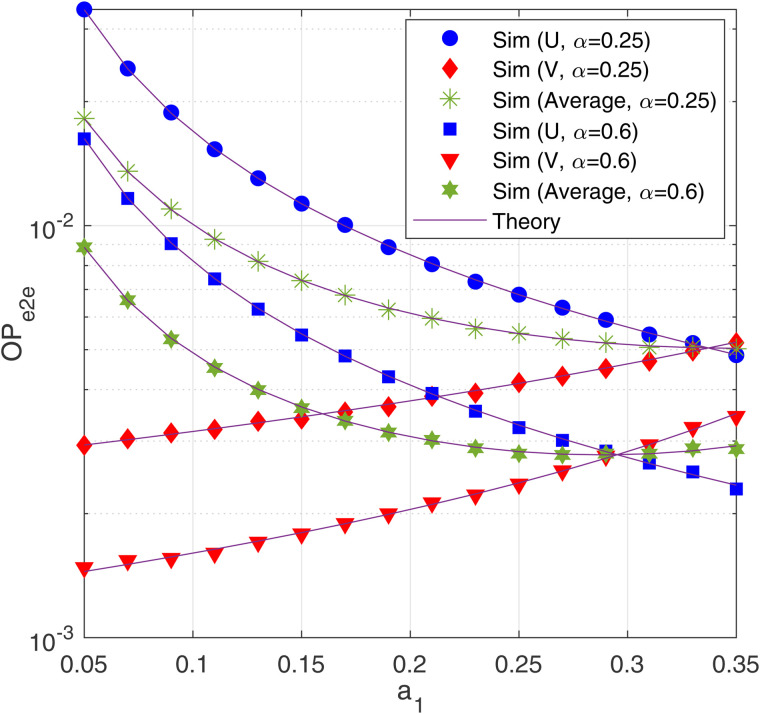
OP-e2e as a function of *α*_1_ with Δ_B_ = 12.5 (dB), *M* = 3 and *N* = 4.

In Fig 4, we present the OP-e2e of U and V, and the average OP-e2e as a function of *a*_1_, with α=0.4 and 0.75, and with $\Delta_{B}$ = 12.5 (dB), *M* = 3 and *N* = 4. As observed, the OP-e2e of V (U) increases (decreases) as *a*_1_ increases. It is worth noting that there exist certain values of *a*_1_ at which the OP-e2e of U and V is the same, which obtains the performance fairness between U and V. It is also seen from Fig 4 that the OP-e2e of U can be lower that of V when *a*_1_ takes high values. Next, we can observe that there are optimal values of *a*_1_ which minimize the average OP-e2e. In particular, as α=0.25 and 0.6, the optimal values of *a*_1_ are 0.31 and 0.29, respectively.

**Fig 5:**
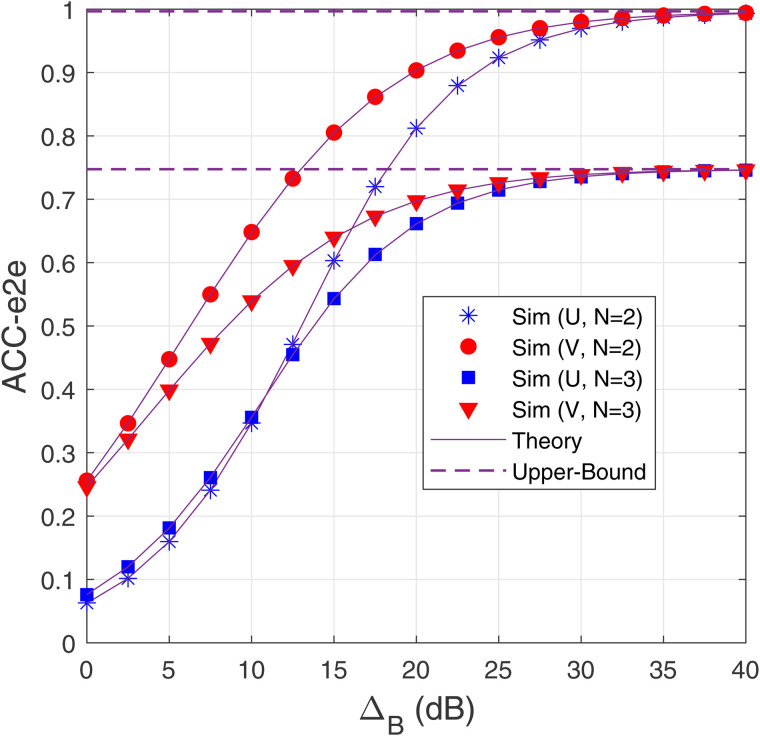
ACC-e2e as a function of Δ_B_ (dB) with *M* = 3, *α* = 0.1 and *α*_1_ = 0.1.

Fig 5 presents the ACC-e2e at U and V as a function of $\Delta_{B}$ (in dB) with *M* = 3, α=0.1, *a*_1_ = 0.1 and *N* = 2,3. As observed, V achieves a higher ACC-e2e than U, and ACC-e2e of both destinations increases as $\Delta_{B}$ increases. However, as $\Delta_{B}$ is sufficiently high, the ACC-e2e of U and V converges to an upper-bound value that is independent of $\Delta_{B}$. Indeed, from (6), (7) and (8), it is straightforward that


$$\psi_{T_{n-1}T_{n}^{\left(b\right)}}^{p_{V}}\overset{\Delta_{B}\to +\infty }{\approx }\frac{a_{2}}{a_{1}},\psi_{T_{N}U^{\left(b\right)}}^{p_{U}}\overset{\Delta_{B}\to +\infty }{\approx }\frac{a_{2}}{a_{1}},\psi_{T_{N}V^{\left(b\right)}}^{p_{V}}\overset{\Delta_{B}\to +\infty }{\approx }\frac{a_{2}}{a_{1}}.$$ (45)

Then, from (45), we have


$$\psi_{U}^{e2e}\overset{\Delta_{B}\to +\infty }{\approx }\frac{a_{2}}{a_{1}},\psi_{V}^{e2e}\overset{\Delta_{B}\to +\infty }{\approx }\frac{a_{2}}{a_{1}},$$ (46)

which leads to


$${ACC}_{e2e}^{X}\overset{\Delta_{B}\to +\infty }{\approx }\left(1-\alpha \right)\tau \log_{2}\left(1+\frac{a_{2}}{a_{1}}\right),\;\;X\in \left\{U,V\right\}.$$ (47)

It is also seen from Fig 5 that at medium and high $\Delta_{B}$ values, the ACC-e2e performance of both destinations improves with lower number of hops (*N* + 1). This is because the time allocated for each hop increases as *N* decreases, thereby enhancing the channel capacity obtained at each hop. Finally, we can observe that the simulations confirm the accuracy of the analysis.

**Fig 6:**
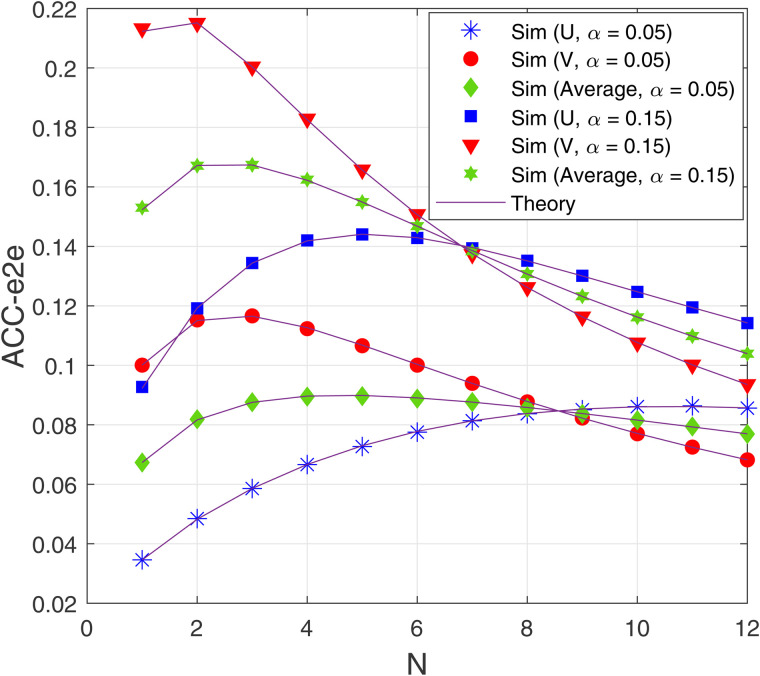
E2e average channel capacity as a function of *N* with Δ_B_ = 0 dB, *M* = 2 and *α*_1_ = 0.2.

Fig 6 analyzes the impact of the number of hops (N) on ACC-e2e of U and V when Δ=0 dB, *M* = 2 and *a*_1_ = 0.2. Since increasing the number of hops (*N*) reduces the distance between two adjacent nodes $T_{n-1}$ and $T_{n}$, it can be observed from Fig 6 that, as *N* increases from 1, the ACC values at both users U and V increase. However, as *N* increases, the time allocated to each hop decreases, leading to a reduction in the instantaneous channel capacity of each hop (see equation (14)). Therefore, it is seen that the ACC values at U and V reduce as *N* is high. To determine the optimal value of *N*, we should consider the average ACC-e2e at U and V, i.e., $\overset{Avg}{\underset{e2e}{ACC}}=\left(\overset{V,Prop}{\underset{e2e}{ACC}}+\overset{V,Prop}{\underset{e2e}{ACC}}\right)/2$. As observed, $\overset{Avg}{\underset{e2e}{ACC}}$ with α=0.15 is highest as *N* = 2, while $\overset{Avg}{\underset{e2e}{ACC}}$ with α=0.05 reaches its highest value at *N* = 4. Moreover, in this figure, $\overset{Avg}{\underset{e2e}{ACC}}$ with α=0.15 is higher than that with α=0.05.

**Fig 7:**
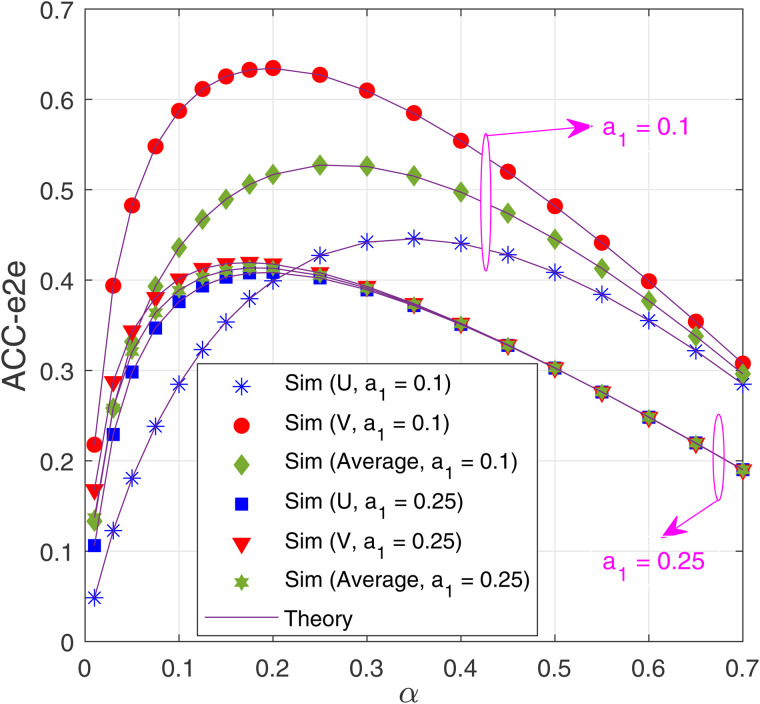
E2e average channel capacity as a function of *α* with Δ_B_ = 10 (dB), *M* = 2 and *N* = 2.

Fig 7 investigates the impact of the time allocation factor α on the ACC-e2e performance as Δ=10 dB, *M* = 2 and *N* = 2. As we can see, the ACC-e2e values vary with changes in α. Notably, with low α, the harvested energy and the transmit power of the transmitters are low, resulting in a low ACC-e2e at both destinations. However, if α is too high, the time allocated for the data transmission is low, which also degrades the ACC-e2e performance. Therefore, as seen from Fig 7, there exist optimal values of α at which ACC-e2e of U and V is highest. For example, with *a*_1_ = 0.1, ACC-e2e of U and V is maximum at α=0.35 and α=0.2, respectively. However, α should be designed to maximize the average ACC-e2e $\begin{array}{c} \left(\overset{Avg}{\underset{e2e}{ACC}}\right) \end{array}$. Indeed, if *a*_1_ = 0.1, the optimal value of α is 0.25. Fig 6 also shows that $\overset{Avg}{\underset{e2e}{ACC}}$ with *a*_1_ = 0.1 is higher than $\overset{Avg}{\underset{e2e}{ACC}}$ with *a*_1_ = 0.25. However, as *a*_1_ = 0.1, the performance gap between U and V becomes higher.

**Fig 8:**
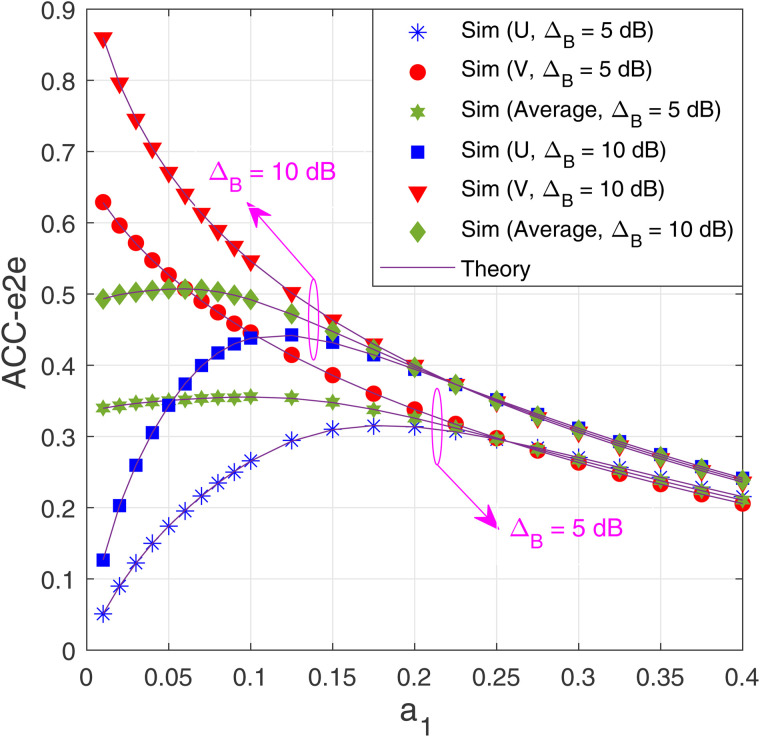
E2e average channel capacity as a function of *α*_1_ with *α* = 0.2, *M* = 3 and *N* = 3.

In Fig 8, the impact of the power allocation $\left(a_{1}\right)$ on the ACC-e2e performance of both destinations is investigated. In this simulation, we set α=0.2, *M* = 3 and *N* = 3. It is seen from Fig 7 that ACC-e2e of V always decreases with the increasing of *a*_1_ because the power allocated to the data $p_{V}$ decreases (or the value of *a*_2_ decreases). For the destination U, we see that there exists an optimal value of *a*_1_ at which ACC-e2e is highest. For example, as $\Delta_{B}=$ 5 dB ($\Delta_{B}=$ 10 dB), the optimal value of *a*_1_ is 0.175 (0.125). However, as mentioned in Fig 7, we should design the value of *a*_1_ to maximize the average ACC-e2e ($\overset{Avg}{\underset{e2e}{ACC}}$). Indeed, Fig 7 shows that with $\Delta_{B}=$5 dB ($\Delta_{B}=$10 dB), $\overset{Avg}{\underset{e2e}{ACC}}$ achieves its maximum value when $\begin{array}{c} a_{1}=0.1\left(a_{1}=0.06\right) \end{array}$. However, it is worth noting that when *a*_1_ is low, the performance gap between U and V is too high.

## Conclusion

In this paper, we derived closed-form expressions of the OP-e2e and ACC-e2e for the proposed NOMA-based multi-hop SIMO scheme using FCs and RF-EH over Rayleigh fading channels. These expressions were validated through Monte Carlo simulations. The results demonstrated that the far destination (V) generally outperforms the near user (U), with a significant performance gap when the transmit SNR $\begin{array}{c} \left(\Delta_{B}\right) \end{array}$, number of hops $\begin{array}{c} \left(N\right) \end{array}$, the time allocation factor $\begin{array}{c} \left(\alpha \right) \end{array}$, and the power allocation factors $\begin{array}{c} \left(a_{1},a_{2}\right) \end{array}$ are low. Moreover, we proved that at high $\Delta_{B}$ region, the ACC-e2e of both destinations converges to an upper-bound value which is independent of $\Delta_{B}$. Additionally, the findings revealed that the OP-e2e and the ACC-e2e can be optimized by appropriately selecting the system parameters such as *N*, $\begin{array}{c} \alpha \end{array}$ and *a*_1_. Finally, the OP-e2e and ACC-e2e performance can be further improved by increasing the number of receiving antennas at the nodes $\begin{array}{c} \left(M\right) \end{array}$.

Our future work will concentrate on jointly optimizing the key parameters—the energy harvesting duration ($\begin{array}{c} \tau \end{array}$) and the number of relays (*N*)—to maximize system capacity by effectively managing inherent system trade-offs. Building on this foundation, we will extend the framework to incorporate non-ideal practical constraints, such as synchronization errors and hardware imperfections, and formulate a rigorous multi-objective optimization problem. Finally, we plan to explore advanced applications by integrating the system into heterogeneous networks and leveraging Machine Learning techniques for dynamic resource management.
